# Comparison of Transverse Mandibular Anatomy in Class III Patients With and Without Cleft Lip and Palate

**DOI:** 10.21142/2523-2754-1303-2025-249

**Published:** 2025-08-31

**Authors:** Marina de Almeida Barbosa Mello, Bhárbara Marinho Barcellos, Isabela Toledo Teixeira da Silveira, Ana Carolina Bonetti Valente, Luciano Reis de Araújo Carvalho, Maria Carolina Malta Medeiros, Renato Yassutaka Faria Yaedú

**Affiliations:** 1 Bauru School of Dentistry, University of São Paulo, Bauru. São Paulo, Brasil. dramarinamello.buco@gmail.com, isabelattsilveira@gmail.com, lucianoreisc@gmail.com, renatoyaedu@gmail.com Universidade de São Paulo Bauru School of Dentistry University of São Paulo São Paulo Brazil dramarinamello.buco@gmail.com isabelattsilveira@gmail.com lucianoreisc@gmail.com renatoyaedu@gmail.com; 2 Craniofacial Anomalies Rehabilitation Hospital, University of São Paulo. Bauru, São Paulo, Brasil. bharbarambarcellos@hotmail.com, anacarolina-valente@alumni.usp.br, carolinamaltamedeiros@gmail.com Universidade de São Paulo Craniofacial Anomalies Rehabilitation Hospital University of São Paulo São Paulo Brazil bharbarambarcellos@hotmail.com anacarolina-valente@alumni.usp.br carolinamaltamedeiros@gmail.com

**Keywords:** cleft lip and palate, mandibular anatomy, orthognathic rurgery, labio y paladar hendido, anatomía mandibular, cirugía ortognática

## Abstract

**Objective:**

This study aimed to analyze and compare mandibular anatomy in Class III patients with and without cleft lip and palate (CLP) to assess anatomical differences and their potential impact on fracture risks during orthognathic surgery.

**Material and Methods:**

Cone beam computed tomography (CBCT) scans were used to analyze 300 hemimandibles from two groups: a control group of non-CLP individuals (n=150) and a CLP group (n=150). Mandibular cross-sectional types were assessed, focusing on height, angulation, and fossa depth in the molar region. Measurements were made at standardized locations to ensure consistency. Statistical analysis was performed using independent t-tests to compare the two groups.

**Results:**

The CLP group demonstrated greater mandibular height (mean 25.6 mm) compared to the control group (mean 25.3 mm), although no significant difference was observed between the groups (p > 0.05). Mandibular angulation was also higher in the CLP group (mean 69.99°) than in the control group (mean 66.35°), with a significant difference (p < 0.05). However, no significant difference was observed in fossa depth between the two groups, with the CLP group measuring 0.64 mm and the control group 0.51 mm (p > 0.05).

**Conclusions:**

The results indicate that CLP may influence mandibular anatomy, particularly in terms of height and angulation, which could affect surgical approaches in procedures like bilateral sagittal split osteotomy. These anatomical variations should be taken into account to reduce fracture risks and improve surgical outcomes for CLP patients undergoing orthognathic surgery.

## RESUMEN

**Objetivo:**

Este estudio tuvo como objetivo analizar y comparar la anatomía mandibular en pacientes con clase III con y sin labio leporino y paladar hendido (LPH), a fin de evaluar las diferencias anatómicas y su posible impacto en los riesgos de fracturas durante la cirugía ortognática.

**Métodos:**

Se utilizaron tomografías computarizadas de haz cónico para analizar 300 hemimandíbulas de dos grupos: un grupo control de individuos sin LPH (n = 150) y un grupo con LPH (n = 150). Se evaluaron los tipos de sección transversal mandibular centrándose en la altura, la angulación y la profundidad de la fosa en la región molar. Las mediciones se realizaron en ubicaciones estandarizadas para asegurar la consistencia. Se realizó un análisis estadístico utilizando pruebas T independientes para comparar los dos grupos.

**Resultados:**

El grupo de LPH demostró una mayor altura mandibular (media de 25,6 mm) en comparación con el grupo control (media de 25,3 mm), aunque no se observó una diferencia significativa entre ambos grupos (p > 0,05). La angulación mandibular también fue mayor en el grupo LPH (media de 69,99°) que en el grupo control (media de 66,35°), con una diferencia significativa (p < 0,05). Sin embargo, no se observó una diferencia significativa en la profundidad de la fosa entre los dos grupos, que fue de 0,64 mm para el grupo LPH y 0,51 mm para el grupo control (p > 0,05).

**Conclusiones:**

Los resultados indican que el LPH puede influir en la anatomía mandibular, particularmente en términos de altura y angulación, lo que podría afectar los enfoques quirúrgicos en procedimientos como la osteotomía sagital bilateral. Estas variaciones anatómicas deben ser consideradas para reducir los riesgos de fractura y mejorar los resultados quirúrgicos en pacientes con LPH sometidos a cirugía ortognática.

## INTRODUCTION

The Bilateral Sagittal Osteotomy of the Mandibular Ramus (BSOMR) is the most commonly performed technique in orthognathic surgery ^1^. One of the most significant complications associated with this procedure is the inadvertent fracture, often referred to as a "bad split"^2^. Inadvertent fractures may be linked to several factors, including improper saw inclination during osteotomy, incorrect splitting of the fragments, anatomical variations of the mandible ^3^, age, and surgeon experience ^4^. However, there remains controversy regarding the actual influence of these factors, as it may be a combination of them that leads to an inadvertent fracture ^5^^,^^6^. 

In terms of mandibular anatomy, several characteristics are recognized as important risk factors for inadvertent fractures. These include the thickness of the lingual cortical bone between the mandibular canal and the posterior border of the ramus, the thickness of the cortical bone at the posterior border of the ramus, the angulation of the mandibular body, the shapes of the mandibular ramus in the axial plane, and the vertical position of the mandibular canal ^7^.

Parnia et al. ^(^^8^^)^ highlighted considerable anatomical variability in the mandibular body region. Additionally, Aarabi et al. ^(^^7^^)^ conducted a retrospective cohort study demonstrating that anatomical alterations of the mandible may increase the risk of inadvertent fractures during orthognathic surgery. However, there are currently no studies in the literature that analyze the relationship between mandibular anatomy in the molar region and fracture patterns. While there are studies in implant dentistry that utilize computed tomography (CT) to examine mandibular anatomy, revealing a high prevalence of anatomical variations, these findings could also be pertinent to the study of inadvertent fractures in orthognathic surgery ^8^^,^^9^.

To date, the literature has not established mandibular patterns specific to individuals with cleft lip and palate (CLP), nor has it identified the prevalent anatomical patterns. Therefore, the aim of this study is to establish and compare the prevalence of mandibular cross-sectional types in the region between the first and second molars among Class III individuals, both with and without CLP, and to determine the prevalence of specific anatomical characteristics for each group.

## MATERIALS AND METHODS

This observational cross-sectional study involved the analysis of Cone Beam Computed Tomography (CBCT) scans from individuals with and without CLP.

The inclusion criteria for the samples were as follows: scans must have a field of view (FOV) encompassing at least the mandible and maxillary teeth. Participants needed to be over 18 years of age and have both first and second lower molars. Additionally, all individuals included in the study had to present with Angle Class III malocclusion, and the tomographic scans had to provide adequate visualization. Specifically for the cleft group, the presence of a unilateral complete cleft was required.

Conversely, the exclusion criteria included the presence of lesions, such as cysts or tumors, or artifacts in the region of interest that could hinder evaluation or alter the anatomy. Participants were excluded if they lacked either the first or second lower molars, as well as individuals with any type of syndrome or those who had previously undergone mandibular reconstruction.

The tomographic scans were subsequently divided into two groups for analysis. Group I (Control) consisted of 100 mandibular cross-sections obtained from individuals without CLP. In contrast, Group II (CLP) included 200 mandibular cross-sections from individuals with unilateral cleft lip and palate (UCLP).

### Tomography Analysis

All analyses and measurements were conducted by a single examiner. The CBCT scans were imported into Dolphin Imaging 11.8 Premium software, and the "Build X-Rays" tool was utilized. The "Cross Section Lower" tool was employed to isolate the mandible region and position the cross-sectional lines between the molars. The mandibles were oriented so that the occlusal plane was parallel to the ground and perpendicular to the reference line provided by the software. The images were then saved in TIFF format for subsequent measurements and shape determination.

In the parasagittal reformations, the left side was mirrored using Adobe Photoshop CS5 software to facilitate analysis and measurement in the same manner as the right side, with the images named according to the respective sides.

### Mandibular Morphological Classification

**Figure 1:**
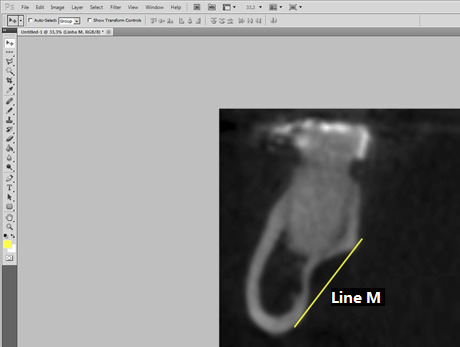
Preparation of the image for fossa measurement. Line M is tangent to the outermost points of the fossa.

**Figure 2:**
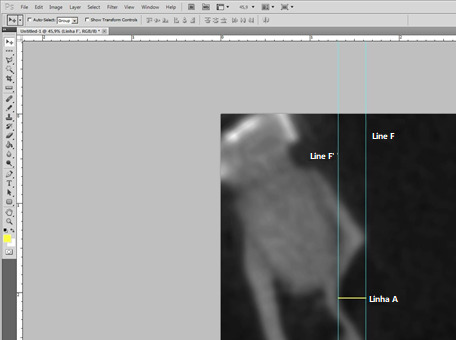
Lines F and F' are tangent to the innermost and outermost points of the fossa, while Line A represents the depth measurement.

Measurements were taken by establishing reference points. The most superior and inferior points of the lingual concavity corresponding to the limits of the mandibular fossa were identified, and a line (Line M) was drawn between them (Figure 1) to delineate the end of the mandibular fossa. The "Straighten" tool in Adobe Photoshop CS5 software was then used to ensure this line was perpendicular to the ground. To visualize this, two additional lines were drawn perpendicular to the ground, marking the beginning (Line F) and end (Line F') of the fossa. The measurement of the mandibular fossa (Line A) (Figure 2) was performed between Lines F and F', ensuring perpendicularity for precise measurements.

The morphological classification was based on the depth of the mandibular fossa, categorized into four types:

• Type a: Distance between 0 and 1 mm
• Type b: Distance between 1.1 and 2 mm
• Type c: Distance between 2.1 and 3 mm
• Type d: Distance greater than 3.1 mm

**Figure 3:**
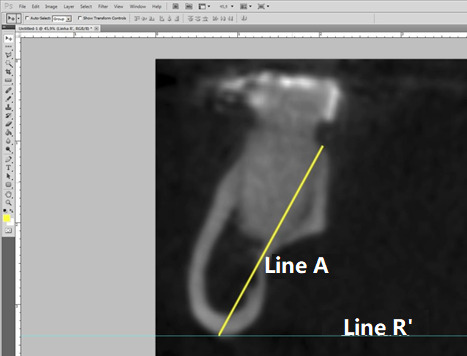
Line R' is tangent to the lowest point of the base of the mandible, while Line B represents the height measurement.

**Figure 4:**
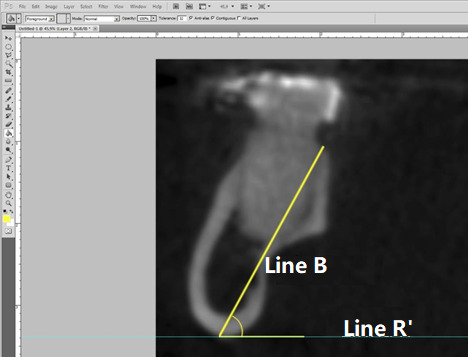
Measurement of the angulation of the mandible between Line R' and Line B.

The height of the mandibles was measured using a line extending from the lingual portion of the alveolar ridge to the lowest point of the mandible base (Figure 3). This line was also used to assess the angulation of the mandible body, defined as the angle formed by Line B and Line R’ (parallel to the ground) (Figure 4).

## STATISTICAL ANALYSIS

All data were tabulated in a Google Sheets spreadsheet and organized for statistical analysis using Sigma Plot 12.0 software. Intra-examiner calibration was conducted using 30% of the sample from each group, with measurements repeated after a 15-day interval. The intraclass correlation coefficient was calculated in Google Sheets to assess method error.

The analysis of the results was divided into two steps. The first step involved comparing the control and CLP groups, as well as the subdivisions of the mandibular fossa between these groups. The second step included tests for distribution and correlation among the variables.

For comparisons between the groups and subdivisions of the mandibular fossa, the Student's t-test was employed when the samples exhibited a normal distribution, while the Mann-Whitney test was used when normality was not met. For correlation tests among the variables within each group and subdivision of the mandibular fossa, Pearson's correlation test was applied.

Lastly, to evaluate the distribution of the mandibular fossa subdivisions, both the Chi-square test and the Mann-Whitney test were utilized. A significance level of 5% was set for all statistical tests.

## RESULTS

For this study, a total of 413 cone beam computed tomography (CBCT) scans were evaluated. Among these, 163 scans were from individuals with CLP, and 100 exams were selected for the study. In the control group, 250 scans were assessed, with 57 exams selected. This number was necessary to complete the analysis of 100 hemimandibles.

From the 163 scans in the CLP group, 63 were excluded due to the absence of molars (5 scans), syndromic patients (2 scans), bilateral clefts (43 scans), or post-foramen conditions (13 scans). In the control group, out of 250 scans, 193 were excluded for not being classified as Class III (182 scans) or for lacking one of the lower molars (11 scans).

Thus, the final sample for this study consisted of 300 hemimandibles, with 100 from the control group and 200 from the CLP group. A sample power analysis was conducted using the depth of the mandibular fossa as the primary variable of interest. This analysis considered a mean difference of 0.5 mm, a standard deviation of 0.877, and an alpha level of 5%, resulting in a sample power of 0.996.

Intra-examiner calibration was performed on a total of 90 hemimandibles (30 from the control group and 60 from the CLP group). The intraclass correlation coefficient (ICC) test indicated a coefficient of 0.99 for all variables, demonstrating satisfactory calibration of the examiner.

Table 1 presents the classification of mandibular types for both the cleft lip and palate (CLP) group and the control group, including the quantity and percentage of each type. In the CLP group, Type a was identified in 37 individuals (18.5%), Type b in 87 individuals (43.5%), Type c in 59 individuals (29.5%), and Type d in 17 individuals (8.5%). In comparison, the control group displayed a slightly different distribution: Type a was present in 13 individuals (13%), Type b in 47 individuals (47%), Type c in 33 individuals (33%), and Type d in 7 individuals (7%). Overall, Type b was the most predominant in both groups, while Type d was the least common. The data highlight the similarities and differences in mandibular type distribution between individuals with CLP and those in the control group.

**Table 1:** Classification of mandibular types for the cleft lip and palate (CLP) group and the control group, including the quantity and percentage of each type.

Type	Cleft (n)	% Cleft	Control (n)	% Control
a	37	18,5	13	13
b	87	43.5	47	47
c	59	29.5	33	33
d	17	8,5	7	7

The mandibular measurements of all mandible types are summarized in Table 2, for both groups. Within the CLP group, the distribution of mandibular types and mandibular measurements between the side with CLP and the side without CLP was also noted and quantified (Table 3).

**Table 2:** Mean and median of the variables height, depth of the fossa, and mandibular angulation for each mandibular type in the cleft lip and palate (CLP) group and the control group.

	Cleft Group		Control group	
	Mean	SD	Mean	SD
Mandibular Type a				
Height (mm)	25.603	4.67	25.315	3.97
Depth (mm)	0.635	0.3	0.508	0.38
Angulation	69.986	5.84	66.346	4.90
Mandibular Type b				
Height (mm)	25.543	3.27	24.496	3.26
Depth (mm)	1.621	0.29	1.526	0.26
Angulation	69.590	4.06	67.360	5.55
Mandibular Type c				
Height (mm)	25.900	3.11	25.264	2.20
Depth (mm)	2.469	0.27	2.427	0.25
Angulation	69.356	4.69	67.218	4.03
Mandibular Type d				
Height (mm)	26.935	3.59	25.686	4.53
Depth (mm)	3.644	0.46	3.343	0.26
Angulation	69.700	4.98	68.671	10.53

SD: Standard Deviation

**Table 3:** Mean and standard deviation of the variables height, depth of the fossa, and mandibular angulation for each mandibular type on the sides with and without cleft lip and palate (CLP) in the CLP Group.

	Side with cleft		Side without cleft	
	Mean	SD	Mean	SD
Mandibular Type a				
Height (mm)	25.495	5.15	25.717	4.14
Depth (mm)	0.642	0.28	0.628	0.38
Angulation	70.911	6.48	69.011	4.50
Mandibular Type b				
Height (mm)	25.584	3.31	25.498	3.13
Depth (mm)	1.633	0.29	1.607	0.26
Angulation	69.213	4.08	69.993	5.62
Mandibular Type c				
Height (mm)	26.167	3.53	25.675	2.06
Depth (mm)	2.474	0.29	2.466	0.26
Angulation	70.078	4.83	68.747	4.03
Mandibular Type d				
Height (mm)	26.867	2.63	27.012	4.53
Depth (mm)	3.456	0.48	3.800	0.26
Angulation	69.844	5.16	69.537	10.53

SD: Standard Deviation

**Table 4:** Comparison between the cleft lip and palate (CLP) group and the control group concerning the variables of angulation, height, and depth for all mandibular types.

	A	B	C	D
	Cleft versus Control	Cleft versus Control	Cleft versus Control	Cleft versus Control
Angulation	5.02x10-2	8.81x10-3	3.02x10-2	0.485 *
Height	0.844	7.89x10-2	0.297 *	0.480
Depth	0.349 *	0.059 *	0.455 *	0.123

* Mann-Whitney Test

When comparing the studied variables between the cleft lip and palate (CLP) group and the control group, a statistically significant difference was observed in the mandibular angulation (p = 0.00139) and in the height of the mandible (p = 0.0459). However, the depth of the mandibular fossa did not show a statistically significant difference (p = 0.697).

When comparing the studied variables for each mandibular type, no statistically significant differences were observed for types a and d. However, significant differences were noted regarding mandibular angulation for type b (p = 0.00881) and type c (p = 0.0302) (Table 4). Additionally, when comparing the sides with and without cleft in the CLP group, none of the analyzed variables showed statistically significant differences within the mandibular types (Table 5). 

**Table 5:** Comparison of the sides with and without cleft lip and palate (CLP) in relation to the variables of angulation, height, and depth for each mandibular type.

	A	B	C	D
	With Cleft versus Without Cleft	With Cleft versus Without Cleft	With Cleft versus Without Cleft	With Cleft versus Without Cleft
Angulation	0.330	0.374	0.281	0.904
Height	0.888	0.653	0.824 *	0.209
Depth	0.854 *	0.902 *	0.277 *	0.894 *

* Mann-Whitney Test

A positive correlation was found between mandibular angle and mandibular height for the CLP group (p = 0.139x10-3) and for the control group (p = 0.0459). The other correlations performed between mandibular angle and depth of the mandibular fossa, and between mandibular height and depth of the mandibular fossa, did not show a statistically significant difference for both groups. However, it was possible to observe a tendency for a positive correlation for both groups when correlating mandibular height and depth of the mandibular fossa, and in the control group between mandibular angle and depth of the mandibular fossa; regarding the CLP group, this tendency was negative (Table 6).

**Table 6:** Correlations between the variables for the cleft lip and palate (CLP) group and the control group.

	CLP	Control
Height versus Depth	0.0839	0.678
Height versus Angulation	1.789x10-12	1.345x10-11
Angulation versus Depth	0.541	0.428

When correlating the variables used in the study for each mandibular type in both groups (control and CLP), it was observed that there was a positive correlation in all mandibular types except in mandibular type a of the control group when correlating angle with mandibular height. Similarly, when evaluating the control and CLP groups overall, there was also no correlation between angle and mandibular fossa, and between mandibular fossa and mandibular height for all mandibular types except mandibular type c of the control group, which showed a statistically significant difference correlating mandibular angle with depth of the mandibular fossa (Table 7).

**Table 7:** Correlations of the variables for the cleft lip and palate (CLP) group and the control group within each mandibular type.

	A		B		C		D	
	Cleft	Control	Cleft	Control	Cleft	Control	Cleft	Control
Height versus Depth	0.738	0.635	0.201	0.149	0.838	0.301	0.898	0.238
Height versus Angulation	5.06x10-3	0.232	1.17x10-5	5.17x10-7	1.45x10-4	5.25x10-3	2.69x10-3	2.53x10-3
Angulation versus Depth	0.383	0.248	0.796	0.430	0.926	8.33x10-3	0.312	0.389

For the mandibular type a, there is a positive correlation trend in the CLP group between the depth of the mandibular fossa and mandibular height, and between angle and mandibular height, as well as a negative correlation between the depth of the mandibular fossa and mandibular angle. Therefore, the deeper the mandibular fossa, higher is the mandible height; as well the higher the mandibular height, higher the angle. But, the deeper the mandibular fossa, smaller was the angle. Regarding the control group, although there is no statistically significant correlation for this mandibular type, there is a trend of negative correlation between the depth of the mandibular fossa and mandibular height, and between the depth of the mandibular fossa and mandibular angle, as well as a trend of positive correlation between angle and mandibular height.

For mandibular type b, it was observed a tendency of positive correlation between the depth of the mandibular fossa and height in the CLP group, and a tendency of negative correlation between the depth of the fossa and mandibular angle. In control group, there is a negative correlation between angle and mandibular height, and a trend of negative correlation between the depth of the mandibular fossa and mandibular angle, as well as between the depth of the fossa and mandibular height. While, in mandibular type c, there is a positive correlation between angle and mandibular height in the CLP group, and a very discreet trend towards a positive correlation between the depth of the mandibular fossa and mandibular height, and a negative correlation between the depth of the fossa and mandibular angle.

In mandibular type d, both in the CLP group and in the control group, there is a positive correlation between angle and mandibular height, and a tendency towards a positive correlation between the depth of the mandibular fossa and height, and the depth of the fossa and mandibular angle in both groups. Although, in the CLP group, the trend of positive correlation between the depth of the mandibular fossa and mandibular angle is very discreet.

CLP scans were divided into sides, with and without cleft, and each mandibular type was distributed according to its classification. As a result, a positive correlation was observed for all mandibular types on the side with and without cleft between angle and mandibular height, except for the side with cleft in mandibular type a. The other correlations between the variables were not statistically significant (Table 8).

**Table 8:** Correlations of the variables for the sides with and without cleft lip and palate (CLP) within each mandibular type.

	A		B		C		D	
	With cleft	Without cleft	With cleft	Without cleft	With cleft	Without cleft	With cleft	Without cleft
Height versus Depth	0.781	0.440	0.287	0.468	0.934	0.764	0.251	0.402
Height versus Angulation	8.13x10-2	2.83x10-2	2.34x10-3	1.83x10-3	3.81x10-2	3.06x10-3	4.85x10-2	2.76x10-3
Angulation versus Depth	0.576	0.500	0.866	0.657	0.951	0.851	0.937	0.810

In mandibular type a on the side with cleft, there is a trend of negative correlation between the depth of the mandibular fossa and mandibular angle, and between the depth of the fossa and mandibular height. On contrary, on the side without cleft, there is a positive correlation between angle and mandibular height, and a tendency of positive correlation between the depth of the fossa and height, as well as a tendency of negative correlation between the depth of the fossa and mandibular angle.

In mandibular type b, there was a positive correlation between angle and mandibular height on both sides (with and without cleft lip and palate), and a trend of positive correlation between the depth of the mandibular fossa and mandibular height on both sides. There was also a very discreet trend of positive correlation between the depth of the mandibular fossa and mandibular angle on the side with cleft lip and palate, and a negative trend of correlation on the side without cleft lip and palate.

In mandibular type c, in addition to the statistically significant positive correlations between angle and mandibular height on both sides, discreet trends of negative correlation between angle and depth of the mandibular fossa were observed on the side without cleft, as well as a discreet trend of negative correlation between the depth of the mandibular fossa and mandibular height on the side with cleft lip and palate, and a positive trend of correlation on the side without cleft lip and palate.

In mandibular type d, there are positive correlations and trends for both sides in almost all correlations, except for the correlation between angle and depth of the mandibular fossa on the side with cleft, which shows a very discreet trend of negative correlation.

## DISCUSSION

It is known that the behavior of fractures can change depending on the anatomy in the more distal region of the osteotomy ^10^. Therefore, the primary benefit of studying variations in anatomy is to reduce inadvertent fractures and maximize the contact surface between bone segments. Among the various techniques described in the literature ^11^^,^^12^^,^^13^^,^^14^^,^^15^, concerning bilateral sagittal osteotomy with mandibular ramus (OSRM) and segment separation techniques^12,15,16^, each can either increase or decrease the rate of unwanted fractures depending on the anatomical variation of the mandible.

The present study was motivated by observations of BSOMR behavior during separation in orthognathic surgeries. Preoperatively, a higher number of mandibles with a pronounced mandibular fossa were noted in patients with cleft lip and palate (CLP). Postoperatively, these mandibles exhibited a fracture pattern that reduced contact between bone segments. Thus, this study investigates whether the occurrence of inadvertent fractures and reduced contact area correlates with anatomical modifications. A review of the literature revealed no studies relating to mandibular anatomy in patients with CLP or its correlation with OSRM. Therefore, numerous factors concerning mandibular anatomy and sagittal osteotomy techniques warrant further study.

In this study, only the epidemiology of cross-sectional anatomy of the mandible was considered, classifying mandibular body morphology in the region between the first and second molars into four different types. The distribution of mandibular types did not vary between the cleft and control groups, with type B (43.5% CLP/47% Control) being the most prevalent for both, followed by type C (29.5% CLP/33% Control), type A (18.5% CLP/13% Control), and type D (8.5% CLP/7% Control).

The study by Parnia et al. ^(^^8^^)^ classified three mandibular morphologies: Type I, with inferior concavity of less than 2 mm; Type II, with concavity between 2 and 3 mm; and Type III, with concavity greater than 3 mm. The frequency of Type I (20%) was lower than that of Types II (52%) and III (28%). This contrasts with the findings in the present study, which indicated that types A and B (both ≤2 mm depth) were the most prevalent ^8^.

However, the present study yielded results similar to those described by Thomas Jung, who categorized the mandibular fossa from -1.0 mm (convexity) to 5.5 mm (maximum concavity) in 0.5 mm intervals. Jung found that half (50%) of the mandibles had a mandibular fossa measuring up to 2.5 mm; 25% of the sample had depths between 2.5 mm and 3 mm, and 25% exceeded 3 mm ^17^^,^^18^.

No significant differences were observed in mandibular classification based on the depth of the mandibular fossa between the CLP and control groups, nor between mandibular types within each group, or between the sides with and without CLP. This indicates that cleft lip and palate did not correlate with this anatomical feature of the mandible, suggesting that the presence of CLP does not alter mandibular development compared to patients without CLP and does not influence the development of one side of the mandible relative to the other. Furthermore, the depth of the mandibular fossa did not show a statistically significant difference between the CLP and control groups overall, nor for each mandibular type, as well as between the sides with and without CLP.

Conversely, for mandibular height and angulation, a statistically significant difference was found between the CLP and control groups. This relationship was also observed in types B and C for angulation, but not in any type for height, possibly due to masking effects in types A and D, which had fewer hemi-mandibles. Thus, it is suggested that CLP may influence the development of the mandible in terms of angulation and height.

Another notable finding is the absence of a relationship between the dimensions of angulation, height, and depth of the fossa on the side with CLP compared to the side without CLP within each mandibular type. This suggests that the presence of CLP does not cause differential development of one side of the mandible.

It is believed that greater depths of the mandibular fossa may influence the fulcrum of osteotomy separation. If these mandibles present unfavorable morphology, they may be more susceptible to inadvertent fractures and exhibit reduced contact surfaces. In such cases, it may be necessary to deepen the osteotomy or chisel toward the base, surpassing the fulcrum determined by the fossa. The challenges in executing the osteotomy, as noted in Aarabi et al.'s study ^7^, may complement these findings, suggesting that the studied variables can influence osteotomy execution.

In agreement with Aarabi et al. ^7^, the height of the mandible can hinder the execution of horizontal ramus osteotomy, as greater caution is required regarding depth to avoid damaging the mandibular canal, which can lead to increased bleeding and injury to the inferior alveolar nerve. Additionally, greater angulation of the mandibular ramus may complicate the positioning of the saw or drill, which must remain parallel to the vestibular cortex to avoid osteotomies that are excessively thin or entirely facing the lingual cortex, potentially resulting in unwanted fractures or poor adaptation of bone segments during fixation^7^.

Andrade et al. (2015) ^(^^19^^)^ and Wolford & Davis (1990) ^(^^16^^)^ highlighted the importance of understanding the anatomy of the mandibular region, which can determine areas of least resistance and the fracture path during OSRM. In Plooij et al.'s ^20^^)^ study, 51% of fractures occurred as described by Hunsuck, while 33% occurred through the mandibular canal, and 16% followed other patterns. Notably, the fracture pattern was influenced by the depth of the median osteotomy.

Muto et al. ^(^^21^^)^ conducted a series of studies, the first demonstrating the most appropriate location for performing the median osteotomy during OSRM. The second varied the surgical technique, with key modifications including placing the vertical osteotomy vestibularly at the second molar and directing it toward the mandibular angle, along with the use of a separator developed by them ^22^. The third study reported how these fractures occur in the mandibular ramus with the technique described in 2008 ^23^. It is noteworthy that variation in technique alters fracture behavior, with 53% of fractures occurring as described by Hunsuck^13^, 22% directed toward the posterior portion of the mandibular ramus, and 15% being unwanted vestibular fractures in the posterior portion, with no fractures occurring through the mandibular canal.

Both Plooij et al. ^(^^20^^)^ and Muto et al. ^(^^23^^)^ described the behavior of OSRM fractures, with the former employing the classic technique and the latter varying techniques for both osteotomy and segment separation, demonstrating a change in fracture behavior.

A significant number of complications related to OSRM may be influenced by anatomical factors. However, there is a scarcity of studies in the literature correlating changes in technique, anatomical variations, and the occurrence of these complications. In individuals with CLP, such studies are particularly rare, considering that 30% to 50% of this patient group undergo orthognathic surgery as part of their rehabilitation ^24^.

This study initiates an investigation into mandibular anatomical variation in cross-section. Further studies should be conducted to minimize unwanted fractures, maximize contact surfaces between bone segments, reduce intraoperative interferences, and investigate the causes of anatomical variation. Flat and three-dimensional models should be employed for further studies to provide physical explanations for the occurrence of intraoperative complications.

Parnia et al. (2010) ^8^^)^ published an article describing anatomical variations in the mandibular fossa region. They evaluated 100 CT scans of individuals requiring mandibular implants, measuring the deepest area for analysis in all images. Their work involved drawing a line between the most superior and inferior points of the lingual concavity corresponding to the mandibular fossa, and a second line was drawn from the deepest point of the fossa to the first, classifying the region into three types: I (lingual concavity less than 2 mm), II (lingual concavity between 2 and 3 mm), and III (lingual concavity greater than 3 mm). Results indicated minimum and maximum depths of 0.4 mm and 6.6 mm, respectively, with 28% of the sample classified as type III.

## CONCLUSION

Based on the employed methodology, it can be concluded that there is no significant difference in the depth of the mandibular fossa between non-cleft patients and those with cleft lip and palate (CLP). However, notable differences were observed in the height and angulation of the mandibular body between these two groups. Additionally, a correlation exists between the height and angulation of the mandible in both non-cleft patients and those with CLP.

Furthermore, the distribution of mandibular types is similar across both groups, including between the sides with and without CLP. Specifically, type B was the most prevalent in both non-cleft patients and patients with CLP. Nonetheless, there is considerable variability in mandibular morphology among these patients, particularly concerning angulation.

This study underscores the importance of understanding these anatomical differences and their implications for surgical planning and outcomes in patients with CLP.
